# Differentiation between closely-related *Impatiens* spp. and regional biotypes of *Impatiens glandulifera* using a highly-simplified and inexpensive method for MALDI-TOF MS

**DOI:** 10.1186/s13007-018-0323-6

**Published:** 2018-07-16

**Authors:** Michael A. Reeve, Kathryn M. Pollard, Daisuke Kurose

**Affiliations:** CABI, Bakeham Lane, Egham, Surrey TW20 9TY UK

**Keywords:** Matrix-assisted laser-desorption and ionisation time-of-flight mass spectroscopy, *Impatiens* species, Himalayan balsam, Plant-biotype discrimination

## Abstract

**Background:**

Matrix-assisted laser-desorption and ionisation time-of-flight mass spectroscopy (MALDI-TOF MS) is a powerful tool for the characterisation and/or identification of protein-containing samples. Several MALDI-TOF MS sample-preparation methods are currently available but few of these are well suited to the analysis of plant material. We have recently developed a simple, rapid, and relatively-cheap method for MALDI-TOF MS that is applicable to plant material (in addition to microbial and insect material), and our aim in this study was to distinguish between closely-related plant species and/or between regional biotypes within an invasive weed species using this method with a view to optimising the selection of biological control agents that can be used for weed management.

**Results:**

We have employed a combination of principal-component analysis and closest-relatedness diagrams derived from MALDI-TOF MS spectral-comparison data to discriminate between the closely-related *Impatiens* spp. *Impatiens noli*-*tangere*, *Impatiens parviflora*, *Impatiens scabrida*, *Impatiens balsamina*, and two regional biotypes of the invasive weed *Impatiens glandulifera*. We have also developed a method for sample discrimination based upon comparison between blind-test MALDI-TOF MS spectra and reference-sample spectra. Using this latter method, we have been able to discriminate on the basis of the acid-soluble-protein mass spectra generated between four regional biotypes of *I. glandulifera* that differ in their susceptibility to the biological control agent Himalayan balsam rust (*Puccinia komarovii* var. *glanduliferae*) using mature leaf material. Using younger leaves, discrimination was not possible between these four regional biotypes.

**Conclusions:**

MALDI-TOF MS analysis is able to discriminate between closely-related plant species within the genus *Impatiens* and between regional biotypes of *I. glandulifera*. Because of this, MALDI-TOF MS holds great promise for improving weed biological control, a management technique which uses highly-specific co-evolved natural enemies for the control of an invasive non-native plant species, through the optimal matching of biological control agents with susceptible target species/regional biotypes.

**Electronic supplementary material:**

The online version of this article (10.1186/s13007-018-0323-6) contains supplementary material, which is available to authorized users.

## Background

Matrix-assisted laser-desorption and ionisation time-of-flight mass spectroscopy (MALDI-TOF MS) employs MALDI soft ionisation of biological samples [1]. In this process, large proteins can be prepared intact in the gas phase with predominantly a single positive charge [2]. The time-of-flight of a charged protein along a tube held at high vacuum after acceleration in an electrical field is proportional to the square root of the mass-over-charge ratio for the protein, thereby allowing a mass spectrum to be generated from the time-of-flight values for the protein components in a particular biological sample. The mass spectrum of a subset of the expressed proteome of a biological sample (normally the highly-expressed acid-soluble proteins, including many ribosomal proteins) is a versatile and sensitive tool for the characterisation and identification of the sample.

MALDI-TOF MS has been used extensively for the identification and/or characterisation of biological samples. A key driver has been human clinical microbiology; a field that has been transformed by MALDI-TOF MS-based diagnostics, particularly for bacterial and yeast infections [8]. This area is extensively and comprehensively reviewed by Clark et al. [5], along with the current art regarding methods for sample preparation that are commonly used within clinical settings for microbial diagnostics. Additional microbial methods have been developed for mycobacteria [6] and for yeasts, notably the rapid-extraction methods developed by Fraser et al. [7]. Lagging behind slightly has been the development of methods for MALDI-TOF MS-based identification and/or characterisation of filamentous fungi [4], though this area is catching up steadily, largely due to the development of robust methods commonly referred to as ‘full-extraction’ protocols after the seminal work of Cassagne et al. [3].

Whilst a number of sample-preparation methods are currently available, the most significant of which can be found in the above references [3–8], these methods are not particularly convenient for the analysis of plant material [9]. Plant material cannot, for example, be used for ‘direct-transfer’ protocols [5] because plant material does not adhere adequately to the sample plate. In addition, the more involved full-extraction protocols [3] also present difficulties for working with plant material because plant biomass does not pellet reliably after centrifugation in 70% (v/v) ethanol and the later formic acid lysis step in these protocols is not able to lyse plant material without additional maceration. In order to overcome some of these difficulties, a highly-simplified and inexpensive method for MALDI-TOF MS sample preparation that is applicable to plant-leaf material has been developed. In short, this method lyses cells by maceration in aqueous acetonitrile containing trifluoroacetic acid (TFA), which selectively extracts acid-soluble proteins. Lysis and extraction are carried out in the presence of near-saturated and inexpensive-grade MALDI matrix. The resulting matrix-saturated lysate containing acid-solubilised proteins is then dried down directly onto the MALDI-TOF MS sample plate, overlaid with additional matrix (if required), and analysed. This method has broad applicability for the characterisation and identification of bacteria, fungi (including rust spores), and insects in addition to plants.

*Impatiens glandulifera* (Balsaminaceae), commonly known as Himalayan balsam, is an annual plant native to the foothills of the Himalayas. It was first introduced as a garden ornamental into Kew Gardens, UK in 1839 [10] and has since naturalised and spread throughout the UK to become one of the UK’s most-prevalent invasive species. In 2006, CABI initiated a biological control programme against Himalayan balsam and conducted surveys for natural enemies across the native range [11]. A rust fungus, identified as *Puccinia komarovii* var. *glanduliferae* [12] was prioritised due to its prevalence and high level of damage in the field. Extensive studies using a strain of the rust (IMI 398718) from Kullu Valley, Himachal Pradesh, India were conducted under quarantine conditions and detailed the life-cycle, infection parameters, and host-range. Host-range experiments assessed the susceptibility of 74 plant species towards the rust, and the test-plant list included native, ornamental, and economically-important plant species of relevance to Europe [13]. Closely-related species included *Impatiens balsamina* (an ornamental, rarely grown in the UK), *Impatiens scabrida* (an ornamental), *Impatiens parviflora* (an invasive species widespread across the UK and Europe) and *Impatiens noli*-*tangere* (a UK native with high conservation importance [14]). The rust was found to be highly host-specific, and only *I. glandulifera* and *I. balsamina* were fully susceptible to the rust [13].

The culmination of this research formed a Pest Risk Assessment (PRA) and, following official government approval, the Indian strain of the rust was released in England in September 2014 [15]. Subsequent field releases across England and Wales in 2015 and 2016 revealed that regional biotypes of *I. glandulifera* differed in their susceptibility towards the rust, with some regional biotypes being fully susceptible, whilst others are resistant [16]. This, combined with results reported by Nagy and Korpelainen [17], who found that *I. glandulifera* was introduced into the UK multiple times from different locations in the native range (both India and Pakistan), strongly suggests that a number of different biotypes of *I. glandulifera* exists throughout the UK. In response to this, a second strain of the rust (IMI 505791), from Kaghan Valley, Khyber Pakhtunkhwa Province, Pakistan was retrieved from storage in liquid nitrogen and its host-specificity was confirmed through the testing of a number of closely-related species [16]. Initial assessments found that the Pakistan strain can infect a significant number of *I. glandulifera* regional biotypes that are resistant to the Indian strain. Permission to release this strain was granted in January 2017 and, after conducting susceptibility assessments for each regional biotype, the most virulent strain of the rust was released at field sites during 2017.

With the aim of providing a rapid and inexpensive method for analysing the relatedness between plant samples that could be used to tailor these rust-based biological control agents to target-species plants and susceptible plant biotypes within the target species faster and cheaper than our current method (growing plants and then testing them empirically by inoculating with different rust strains), we have employed the above MALDI-TOF MS-based method to differentiate between closely-related *Impatiens* spp. and between regional biotypes of the invasive species *I*. *glandulifera*. The closely-related *Impatiens* spp. were chosen based upon prior knowledge of their relatedness from phylogenetic analysis of barcoding data [18]. The regional biotypes for *I. glandulifera* were chosen on the basis of their availability and their differing susceptibility to the two strains of Himalayan balsam rust, thus providing a convenient testbed for our MALDI-TOF MS-based approach to tailor specific rust strains to regional invasive-weed biotypes that have differing susceptibilities. In our study, we used three iterations of experimental design and data analysis for sample discrimination, the benefits and limitations of which will be discussed.

## Methods

### Plant growth

Seeds were sourced from field collections in the UK during 2016 and were stored for a period of at least 6 months at 4 °C before use. Following storage, seeds were placed on sterile filter paper moistened with sterile distilled water in a 9-cm diameter Petri dish, transferred to a 4 °C incubator, and observed every 5 days for signs of germination. Following germination, seedlings were potted on in 10-cm diameter plant pots containing approximately 200 g of John Innes compost (Westland, Sandbach, UK). Plants were maintained in a glasshouse growth room at 21 °C with natural lighting for a period of 12 weeks before sampling of leaf material.

### Samples

For discrimination between closely-related *Impatiens* spp. and regional biotypes of *I*. *glandulifera* using grouping based on principal-component analysis (PCA) and closest-relatedness diagrams (Experiment 1), samples were collected from glasshouse-grown: *I*. *glandulifera* originating from Harmondsworth Moor and Silwood Park, UK; *I*. *noli*-*tangere*; *I*. *parviflora*; *I*. *scabrida*; and *I*. *balsamina*. One leaf was sampled per plant of each species and excised to four fragments of roughly 2 mm × 2 mm (Additional file 1: Figure S1). Samples (numbered 1–24) were randomised and processed blind in numbered Eppendorf tubes for grouping on the basis of MALDI-TOF MS data. For discrimination between regional biotypes of *I*. *glandulifera* by identification of test samples against reference-sample spectra using single leaves from single plants (Experiment 2), samples (numbered 25–40) were collected from glasshouse-grown samples of four UK regional biotypes of *I*. *glandulifera* with different susceptibilities to the India-strain and Pakistan-strain of the rust. The sample regional biotypes originated from Harmondsworth Moor, Middlesex and Lampeter, Ceredigion (India-strain-susceptible, Pakistan-strain-susceptible); Rhosmaen, Carmarthenshire (India-strain-resistant, Pakistan-strain-susceptible); and Silwood Park, Berkshire (India-strain-resistant, Pakistan-strain-partially-susceptible). Three leaf fragments were used to generate reference MALDI-TOF MS spectra from each regional biotype and four leaf fragments were used for ‘blind-testing’ against these reference spectra (Additional file 2: Figure S2). For discrimination between regional biotypes of *I. glandulifera* by identification of test samples against reference-sample spectra using multiple leaves from multiple plants (in order to investigate whether leaf age has an impact upon the ability to discriminate between regional biotypes) (Experiment 3, with samples numbered 41–56), two leaves with different growth stages (an old, mature leaf taken from the first whorl and a young, fully expanded apical leaf) from two plants of the same four regional biotypes of *I. glandulifera* as mentioned above were used. Each leaf was excised to two fragments (A and B) (Additional file 3: Figure S3). For both methods of discrimination between regional biotypes of *I. glandulifera* by identification of test samples against reference-sample spectra, the excised leaf-fragment samples were approximately 3 mm × 3 mm. All the fragments as mentioned above were soaked in 70% (v/v) ethanol (Sigma, Gillingham, UK, with LC–MS-grade water, Fluka, Gillingham, UK) until use. For Experiment 3, duplicate sample preparations (A and B) were carried out from each leaf and duplicates one microlitre aliquots of each sample preparation (1 and 2) were spotted onto the sample plate. The same plants and leaves were also used to provide reference spectra but this time using a single sample preparation and a single spot on the sample plate so that, in order to eliminate statistical bias, all reference spectra would appear in the Bruker identification tables (maximum 20 samples) prior to averaging.

After decanting and evaporating the 70% (v/v) ethanol, leaf fragments in Experiment 1 were macerated in 80 µl of [11 mg/ml ≥ 98% (TLC-grade) HCCA matrix (Sigma, Gillingham, UK) in 65% (v/v) LC-MS-grade acetonitrile (Fluka, Gillingham, UK), 2.5% (v/v) 99% ReagentPlus^®^-grade TFA (Sigma, Gillingham, UK), and 32.5% (v/v) water] (Solution 1) using the blunt end of a plastic inoculating loop. One microlitre of the resulting crude lysate was pipetted onto the sample plate and air dried. This was then overlaid with a further 1 µl of Solution 1 above, air dried, and loaded into a Bruker Microflex spectrometer (Bruker Daltonik, Bremen, Germany), which was run using the standard manufacturer’s settings. For Experiments 2 and 3, leaf fragments were macerated in 60 µl of [12 mg/ml HCCA matrix in 60% (v/v) acetonitrile, 2.5% (v/v) TFA, and 37.5% (v/v) water] (Solution 2) using the blunt end of a plastic inoculating loop. One microlitre of the resulting crude lysate was then pipetted onto the sample plate singly for Experiment 2 and twice (labelled as 1 and 2) as replicates for Experiment 3. After being air dried, these were then overlaid with a further one microlitre of Solution 2 above, air dried again, and loaded into the Bruker Microflex spectrometer.

### Mass spectrometry

Mass spectrometry covering the range 2–20 kDa was carried out using a Bruker Microflex platform (Bruker Daltonik, Bremen, Germany), using a nitrogen laser at 337 nm and an ion-source voltage of 19.98 kV. Calibration was carried out using the manufacturer’s ‘BTS’ controls (*E. coli* proteins supplemented with ribonuclease A and myoglobin), using peaks with masses at 3637.8; 5096.8; 5381.4; 6255.4; 7274.5; 10,300.2; 13,683.2, and 16,952.3 for calibration according to the manufacturer’s instructions. Spectra were acquired using MALDI Biotyper RTC Version 4.0 (Build 19), and database entries were made as single-spectra MSPs using the Bruker Online Client software suite (Version 4.0.19, Bruker Daltonik, Bremen, Germany) using the manufacturer’s settings.

### Data analysis

PCA was carried out on spectra as indicated using the Bruker Online Client software suite using the manufacturer’s settings to generate ordination plots. Closest-relatedness diagrams were constructed by joining any sample numbers that appeared as analyte and best-match after identification. This process generates relationships where a single line denotes one strongest-match linkage and a double line denotes a pair of strongest-match linkages. For average spectral-similarity analysis, reference samples were first used to construct database ‘reference’ mass spectra, which were then used to identify the unknown ‘blind-test’ samples using the Bruker Online Client software suite and using the manufacturer’s settings. Each blind-test sample was identified against all twelve reference spectra. The resulting logarithmic Bruker scores between 0 and 3 were then converted back to linear scores between 0 and 1000. The resulting linearised values for all three reference spectra derived from each regional biotype were averaged and the regional biotype with the highest average figure was selected as the identification call. The percentage clearance value (defined as the percentage gap between the highest average value and the second-highest average value) was then calculated as a crude measure of the identification confidence, with 0–10% clearance arbitrarily defined as low-confidence, 10–20% clearance as reasonable confidence, and better than 20% clearance as high confidence.

## Results

### Blind-test discrimination between closely-related *Impatiens* spp. and regional biotypes of *I*. *glandulifera* using grouping based on PCA and closest-relatedness diagrams

**Fig. 1:**
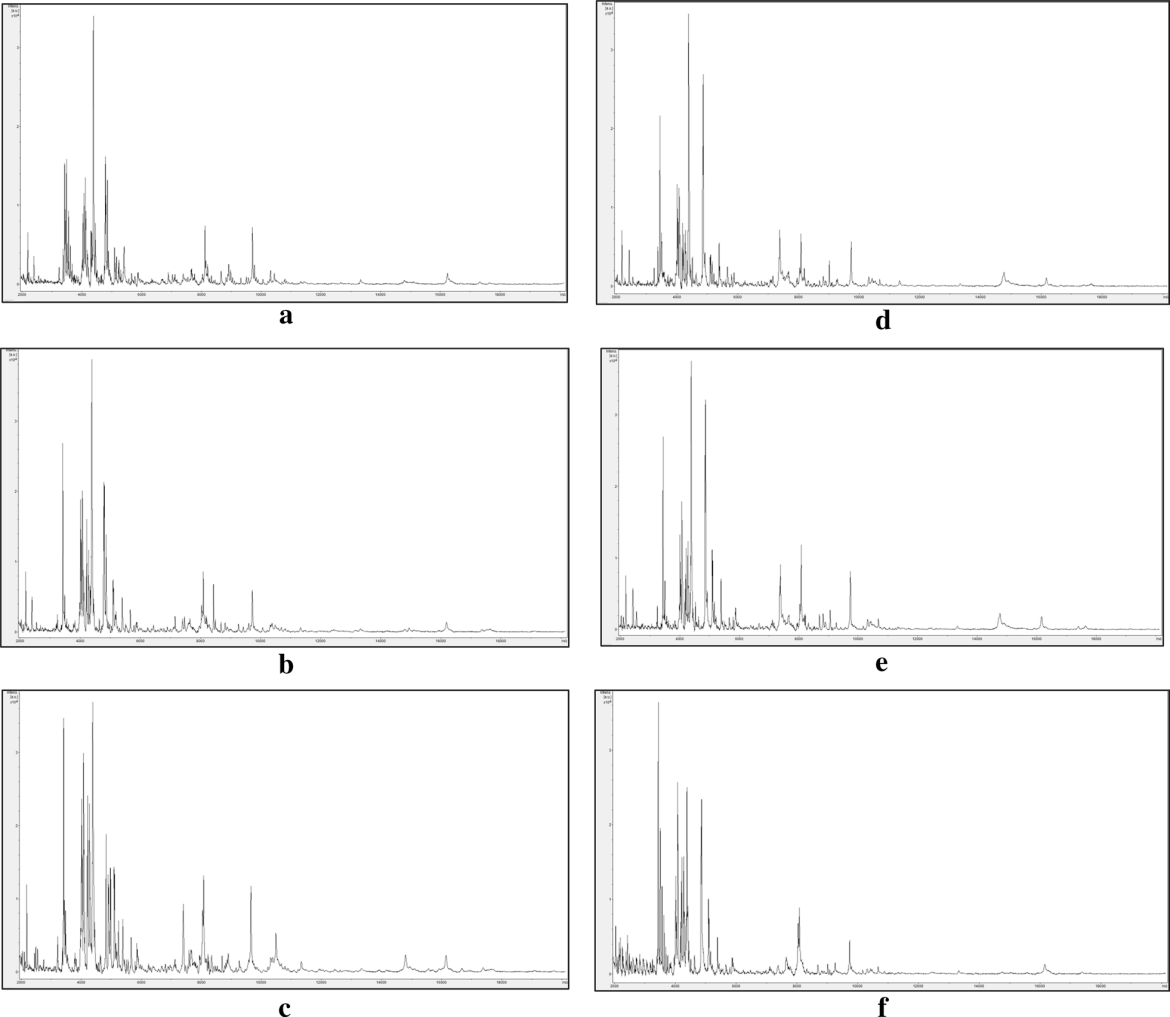
Representative MALDI-TOF MS spectra for leaf samples of **a** *Impatiens noli*-*tangere*, **b** *Impatiens scabrida*, **c** *Impatiens parviflora*, **d**, **e** *Impatiens glandulifera* from **d** Harmondsworth Moor and **e** Silwood Park, and **f** *Impatiens balsamina.* Spectra are shown baseline-subtracted, smoothed, y-axis-autoscaled, and covering the mass range 2–20 kDa

**Fig. 2:**
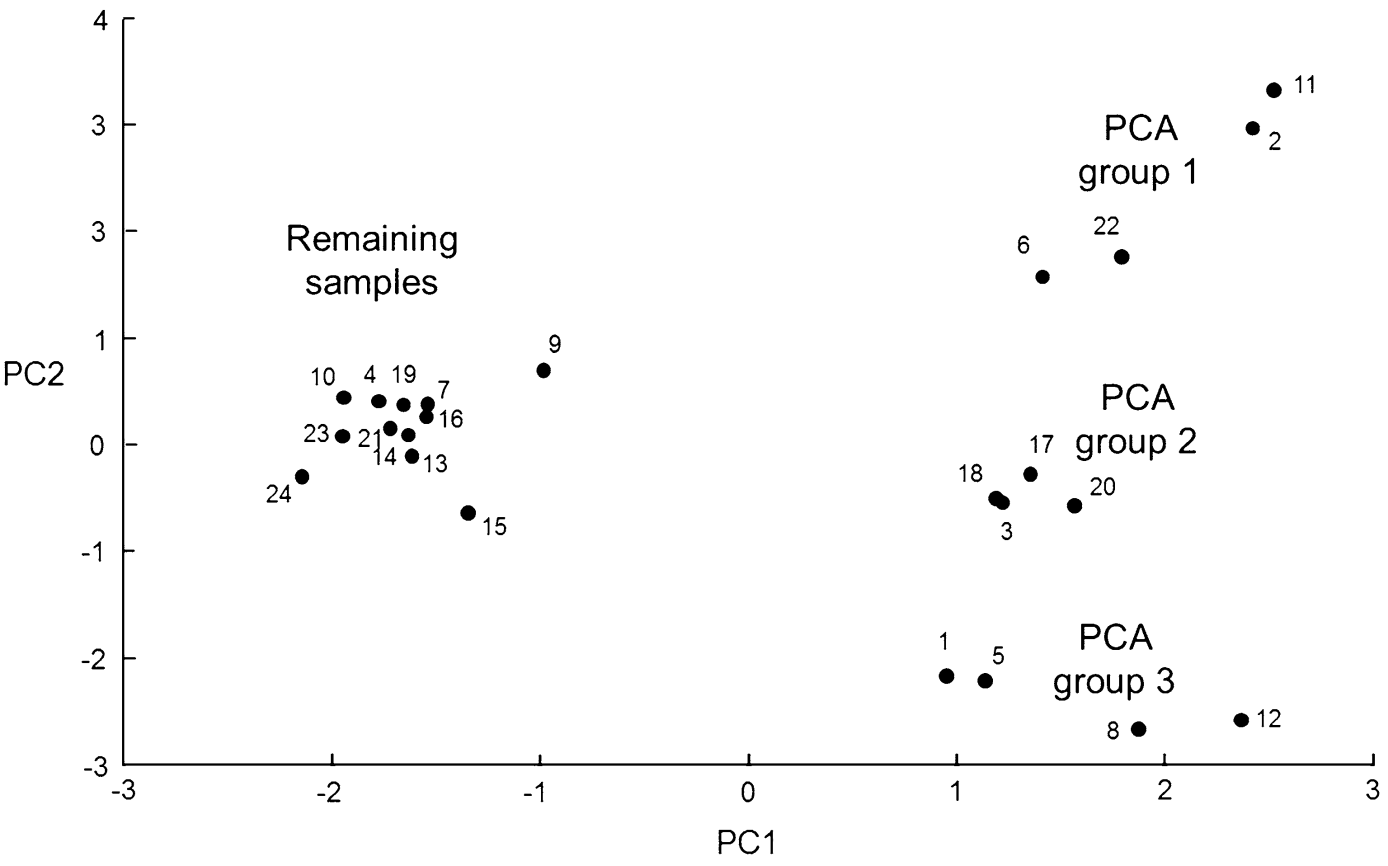
PCA ordination plot derived from the MALDI-TOF MS spectra for blind-test leaf samples of *Impatiens glandulifera* from Harmondsworth Moor and Silwood Park, *Impatiens noli*-*tangere*, *Impatiens parviflora*, *Impatiens scabrida*, and *Impatiens balsamina*

All 24 samples, comprising six groups of closely-related *Impatiens* spp. (including *I*. *noli*-*tangere*, *I*. *parviflora*, *I*. *scabrida* and *I*. *balsamina*) and regional biotypes of *I*. *glandulifera* from Harmondsworth Moor and Silwood Park gave spectra (representative examples of which are shown in Fig. 1) and these were initially analysed blind using PCA to give the ordination plot shown in Fig. 2. On the basis of the blind-test PCA ordination plot, three clear groups of four blind-test samples were observed, along with a more diffuse group of the remaining 12 blind-test samples (Fig. 2). The three distinct groups from the principal-component analysis were PCA group 1 (comprising samples 2, 6, 11, and 22), PCA group 2 (samples 3, 17, 18, and 20), and PCA group 3 (samples 1, 5, 8, and 12). The remaining samples could not readily be grouped on the basis of the PCA ordination plot. Pairwise identifications were therefore carried out between these 24 blind samples using the Bruker Online Client software package. The identification results are given in Table 1.

**Table 1:** Identification scores between closely-related *Impatiens* spp. and regional biotypes of *Impatiens glandulifera*

Blind-test sample number	Best-match blind-test sample number	Bruker score
1	12	2.545
2	11	2.458
3	18	2.384
4	10	2.580
5	8	2.740
6	11	2.508
7	9	2.485
8	5	2.749
9	4	2.475
10	4	2.527
11	2	2.567
12	8	2.623
13	10	2.393
14	21	2.571
15	9	2.220
16	19	2.645
17	20	2.591
18	20	2.568
19	16	2.661
20	17	2.572
21	19	2.564
22	11	2.486
23	4	2.513
24	10	2.562

Closest-relatedness diagrams were then constructed by joining any blind-test sample numbers that appeared in the same row in Table 1. This process generated the relationships shown in Table 2, where a single line denotes one strongest-match linkage in the data from Table 1 and a double line denotes a pair of strongest-match linkages in the data from Table 1. Strongest-match linkage group 1 comprises the same samples as PCA group 3 (samples 1, 5, 8, and 12); strongest-match linkage group 2 comprises the same samples as PCA group 2 (samples 3, 17, 18, and 20); and strongest-match linkage group 3 comprises the same samples as PCA group 1 (samples 2, 6, 11, and 22). Strongest-match linkage group 4 comprises a new and discrete group of four samples (14, 16, 19, and 21) that could not easily be discerned from the PCA ordination plot. Strongest-match linkage group 5 contains complex linkages and the initial resolution prior to unblinding was to split the sample grouping into 4, 9, 10, and 24 and 7, 13, 15, and 23. A better resolution might however have been to take note of the double linkage between samples 4 and 10 and to make 4, 10, 13, and 24 one grouping and the remaining samples (7, 9, 15, and 23) the final grouping by exclusion. The results after unblinding of the test samples are shown in Table 3. PCA group 1 (strongest-match linkage group 3) grouped the *I. parviflora* samples with 100% accuracy, PCA group 2 (strongest-match linkage group 2) grouped the *I. scabrida* samples with 100% accuracy, PCA group 3 (strongest-match linkage group 1) grouped the *I. noli*-*tangere* samples with 100% accuracy, strongest-match linkage group 4 grouped the *I. glandulifera* samples originally collected from Harmondsworth Moor with 100% accuracy, strongest-match linkage group 5 (part) grouped the *I. glandulifera* samples originally collected from Silwood Park with 75% accuracy, and strongest-match linkage group 5 (part) grouped the *I. balsamina* samples with 75% accuracy.

**Table 2:**
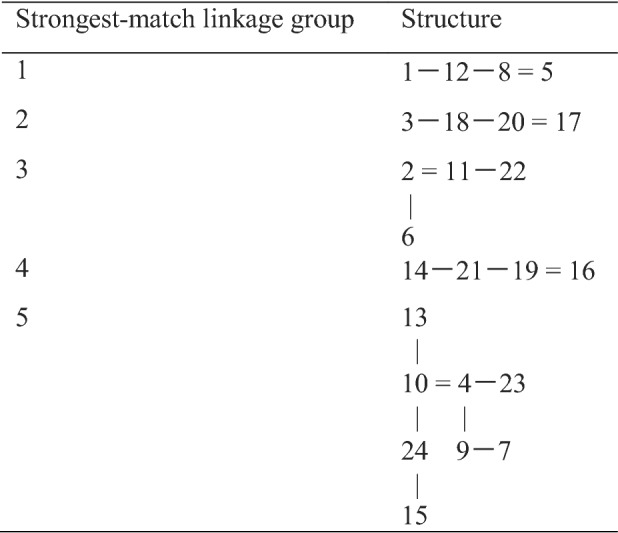
Closest-relatedness diagrams for MALDI-TOF MS spectra for leaf samples of *Impatiens glandulifera* from Harmondsworth Moor and Silwood Park, *Impatiens noli*-*tangere*, *Impatiens parviflora*, *Impatiens scabrida*, and *Impatiens balsamina*

**Table 3:** Groupings for *Impatiens* spp. and biotypes of *Impatiens glandulifera* based on PCA and closest-relatedness diagrams

Test sample number	PCA grouping	Strongest-match linkage group	Identity	Grouping accuracy (%)	Comment
1, 5, 8, 12	3	1	*Impatiens noli*-*tangere*	100	–
3, 17, 18, 20	2	2	*Impatiens scabrida*	100	–
2, 6, 11, 22	1	3	*Impatiens parviflora*	100	–
14, 16, 19, 21	Unclear	4	*Impatiens glandulifera* originally collected from Harmondsworth Moor	100	–
4, 9, 10, 24	Unclear	5 (part)	*Impatiens glandulifera* originally collected from Silwood Park	75	Correct grouping = samples 4, 10, 13, and 24
7, 13, 15, 23	Unclear	5 (part)	*Impatiens balsamina*	75	Correct grouping = samples 7, 9, 15, and 23

### Discrimination between regional biotypes of *I*. *glandulifera* by identification of blind-test test samples against reference-sample spectra using single leaves from single plants

Four regional biotypes of *I*. *glandulifera* with different susceptibilities to the India-strain and Pakistan-strain of Himalayan balsam rust were analysed by MALDI-TOF MS. Averaged linearised identification values for test samples against reference samples and percentage-clearance values are shown in Table 4 for the various blind-test samples (numbered 25–40), with the highest values (the identification calls) indicated in italicised font. The resulting identification calls, with their average confidence ratings were: Harmondsworth Moor (samples 29, 36, 38, and 40, with 27% average clearance); Rhosmaen (samples 25, 30, 33, and 39, with 7% average clearance); Silwood Park (samples 26, 27, 35, and 37, with 26% average clearance); and Lampeter (samples 28, 31, 32, and 34, with 12% average clearance)—all of which were 100% correct upon unblinding.

**Table 4:** Averaged linearised identification values and percentage-clearance values for regional biotypes of *Impatiens glandulifera*

Regional biotype	Blind-test sample number															
	25	26	27	28	29	30	31	32	33	34	35	36	37	38	39	40
Harmondsworth Moor	343	351	165	324	*337*	319	312	311	317	335	310	*383*	301	*302*	259	*360*
Lampeter	179	268	79	*404*	239	188	*366*	*407*	171	*373*	259	180	170	283	150	185
Rhosmaen	*354* ^a^	350	167	304	225	*355*	363	319	*325*	356	254	222	290	204	*298*	245
Silwood Park	316	*449*	*276*	290	225	300	327	273	306	290	*366*	215	*418*	183	247	223
Percentage clearance (%)	3	22	39	20	29	10	1	22	2	5	15	42	28	6	13	32

^a^Highest averaged linearised value (identification call) in each blind-test sample (defined in “Methods” section) shown in italicised font

### Discrimination between regional biotypes of *I*. *glandulifera* by identification of blind-test samples against reference-sample spectra using multiple leaves from multiple plants

**Fig. 3:**
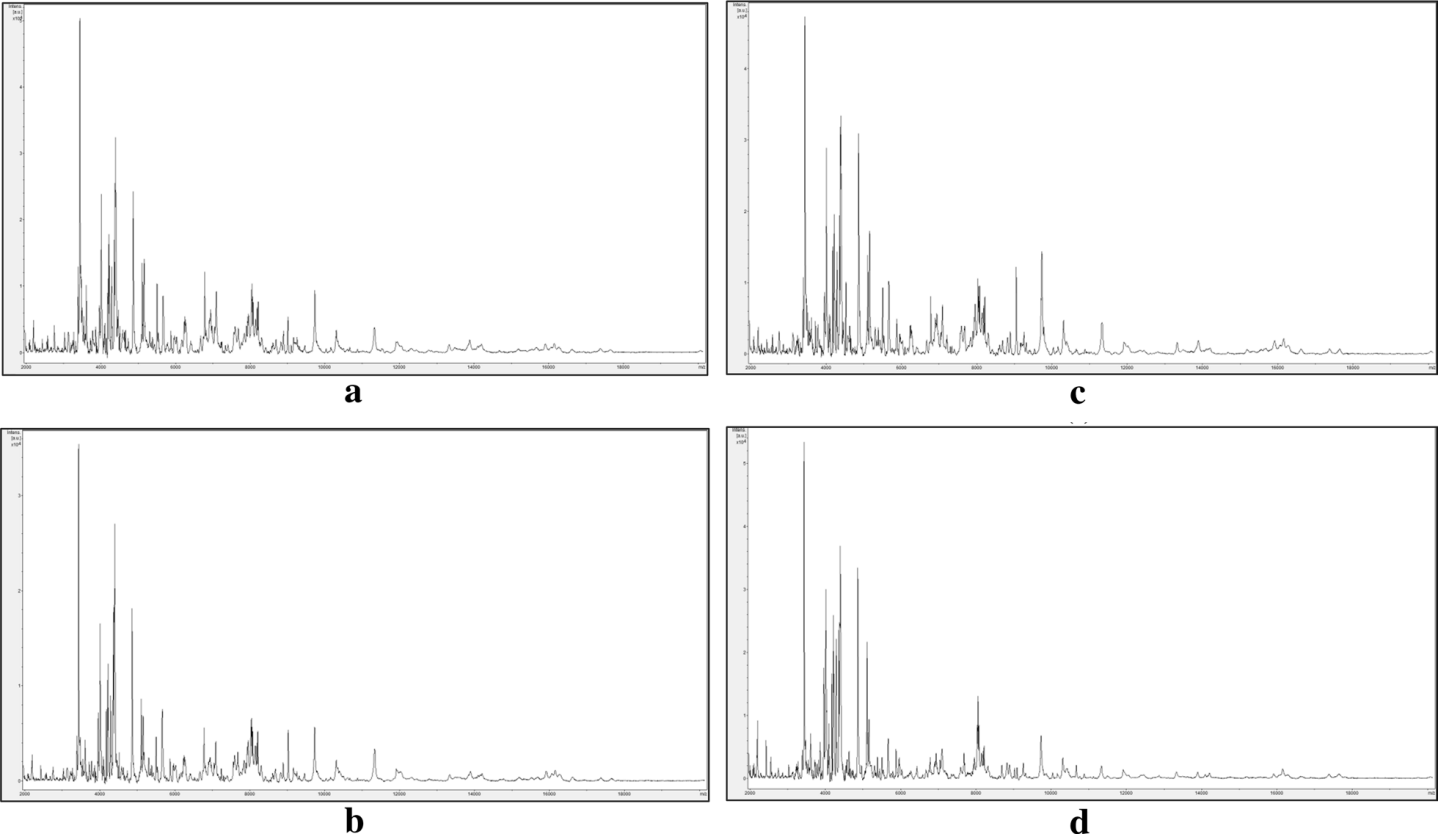
Representative MALDI-TOF MS spectra for leaf samples of *Impatiens glandulifera* from **a** Harmondsworth Moor, **b** Rhosmaen, **c** Silwood Park, and **d** Lampeter. Spectra are shown baseline-subtracted, smoothed, y-axis-autoscaled, and covering the mass range 2–20 kDal

The same four regional biotypes of *I*. *glandulifera* were again analysed by MALDI-TOF MS but this time using two plants from each regional biotype and two leaves with different growth stages from each plant in order to investigate whether leaf age has an impact upon the ability to discriminate between regional biotypes. Representative example spectra are shown in Fig. 3.

The average linearised values obtained for the various old-leaf blind-test samples are shown in Table 5, with the highest values (the identification calls) indicated in italicised font. The identification calls for blind-test samples 41 and 48 are all Silwood Park, the identification calls for blind-test samples 42 and 45 are all Lampeter, the identification calls for blind-test samples 46 and 56 are predominantly Harmondsworth Moor (with blind_test_46_B_1 identified as Silwood Park and blind_test_56_B_2 also identified as Silwood Park), and the identification calls for blind-test samples 47 and 53 are all Rhosmaen. Averaging the A1, A2, B1, and B2 calls gave the old-leaf identification calls indicated in Table 6, with blind-test samples 41 and 48 identified as Silwood Park, blind-test samples 42 and 45 identified as Lampeter, blind-test samples 46 and 56 identified as Harmondsworth Moor, and blind-test samples 47 and 53 identified as Rhosmaen. All of these identifications gave percentage-clearance values in the range 10–20% (‘reasonable confidence’) and all were correct upon unblinding.

**Table 5:** Averaged old-leaf linearised identification values for regional biotypes of *Impatiens glandulifera*

Sample	Harmondsworth Moor	Lampeter	Rhosmaen	Silwood Park
Blind_test_41_A_1	262	364	313	*378*
Blind_test_41_A_2	213	352	323	*378*
Blind_test_41_B_1	363	401	372	*527*
Blind_test_41_B_2	216	328	275	*363*
Blind_test_42_A_1	255	*340*	278	300
Blind_test_42_A_2	258	*354*	286	278
Blind_test_42_B_1	306	*428*	343	386
Blind_test_42_B_2	290	*416*	331	295
Blind_test_45_A_1	263	*413*	294	396
Blind_test_45_A_2	258	*373*	333	350
Blind_test_45_B_1	315	*446*	336	311
Blind_test_45_B_2	313	*432*	414	359
Blind_test_46_A_1	*282* ^a^	146	172	233
Blind_test_46_A_2	*358*	275	284	281
Blind_test_46_B_1	224	197	203	*242*
Blind_test_46_B_2	*324*	274	186	274
Blind_test_47_A_1	227	322	*390*	282
Blind_test_47_A_2	272	297	*365*	322
Blind_test_47_B_1	221	298	*340*	265
Blind_test_47_B_2	231	284	*365*	289
Blind_test_48_A_1	305	272	255	*359*
Blind_test_48_A_2	332	318	310	*373*
Blind_test_48_B_1	384	322	281	*404*
Blind_test_48_B_2	322	341	355	*375*
Blind_test_53_A_1	237	345	*367*	288
Blind_test_53_A_2	314	348	*381*	323
Blind_test_53_B_1	267	350	*384*	336
Blind_test_53_B_2	150	199	*278*	200
Blind_test_56_A_1	*353*	262	255	278
Blind_test_56_A_2	*304*	228	254	288
Blind_test_56_B_1	*266*	176	204	221
Blind_test_56_B_2	234	203	186	*242*

^a^Highest averaged linearised value (identification call) in each blind-test sample (defined in “Methods” section) shown in italicised font

**Table 6:** Old-leaf identification calls and percentage-clearance values

Sample	Identification call^a^	Percentage clearance^b^	Confidence^c^	Unblinding	Correct answer?
Blind_test_41	Silwood	12.0	Reasonable	Silwood	Yes
Blind_test_42	Lampeter	18.1	Reasonable	Lampeter	Yes
Blind_test_45	Lampeter	14.9	Reasonable	Lampeter	Yes
Blind_test_46	Harmondsworth	13.3	Reasonable	Harmondsworth	Yes
Blind_test_47	Rhosmaen	17.7	Reasonable	Rhosmaen	Yes
Blind_test_48	Silwood	11.1	Reasonable	Silwood	Yes
Blind_test_53	Rhosmaen	11.9	Reasonable	Rhosmaen	Yes
Blind_test_56	Harmondsworth	11.1	Reasonable	Harmondsworth	Yes

^a,b,c^Defined in “Methods” section

The average linearised values obtained for the various young-leaf blind-test samples are shown in Table 7, with the highest values (the identification calls) again indicated in italicised font. The identification calls for blind-test samples 43, 49, and 51 are all Harmondsworth Moor; the identifications calls for blind-test sample 50 are all Rhosmaen; the identifications calls for blind-test sample 54 are all Lampeter; the identifications calls for blind-test sample 44 are split between Harmondsworth Moor (two samples), Lampeter (one sample), and Rhosmaen (one sample); the identifications calls for blind-test sample 52 are split between Lampeter (two samples) and Rhosmaen (two samples); and the identifications calls for blind-test sample 55 are split between Lampeter (three samples) and Rhosmaen (one sample). Averaging the A1, A2, B1, and B2 calls gave the young-leaf identification calls indicated in Table 8, with blind-test samples 43, 44, 49, and 51 identified as Harmondsworth Moor; blind-test samples 50 and 52 identified as Rhosmaen; and blind-test samples 54 and 55 identified as Lampeter. Upon unblinding, samples 43, 49, 50, 52, and 55 were correctly identified whilst samples 44, 51, and 54 were incorrectly identified. This suggests that, using older leaves, it is possible to distinguish between the above four regional biotypes but, for younger leaves, the results are less reliable.

**Table 7:** Averaged new-leaf linearised identification values for regional biotypes of *Impatiens glandulifera*

Sample	Harmondsworth	Lampeter	Rhosmaen	Silwood
Blind_test_43_A_1	*347* ^a^	219	196	206
Blind_test_43_A_2	*251*	156	170	184
Blind_test_43_B_1	*388*	239	208	203
Blind_test_43_B_2	*493*	291	224	220
Blind_test_44_A_1	*335*	239	201	222
Blind_test_44_A_2	*329*	206	193	182
Blind_test_44_B_1	282	*285*	237	251
Blind_test_44_B_2	270	263	*271*	269
Blind_test_49_A_1	*325*	131	212	182
Blind_test_49_A_2	*387*	239	218	197
Blind_test_49_B_1	*330*	283	236	221
Blind_test_49_B_2	*320*	243	189	219
Blind_test_50_A_1	265	323	*363*	204
Blind_test_50_A_2	253	322	*355*	187
Blind_test_50_B_1	272	308	*357*	243
Blind_test_50_B_2	237	346	*389*	202
Blind_test_51_A_1	*300*	234	213	184
Blind_test_51_A_2	*343*	230	211	154
Blind_test_51_B_1	*296*	223	192	191
Blind_test_51_B_2	*265*	219	206	134
Blind_test_52_A_1	218	334	*401*	222
Blind_test_52_A_2	146	306	*327*	211
Blind_test_52_B_1	112	*203*	139	102
Blind_test_52_B_2	262	*300*	286	195
Blind_test_54_A_1	178	*289*	288	244
Blind_test_54_A_2	227	*345*	279	272
Blind_test_54_B_1	232	*293*	238	272
Blind_test_54_B_2	204	*255*	248	228
Blind_test_55_A_1	171	176	*230*	147
Blind_test_55_A_2	222	*282*	235	214
Blind_test_55_B_1	246	*341*	278	236
Blind_test_55_B_2	205	*300*	239	208

^a^Highest averaged linearised value (identification call) in each blind-test sample (defined in “Methods” section) shown in italicised font

**Table 8:** Young-leaf identification calls and percentage-clearance values

Sample	Identification call^a^	Percentage clearance^b^	Confidence^c^	Unblinding	Correct answer?
Blind_test_43	Harmondsworth	39	High	Harmondsworth	Yes
Blind_test_44	Harmondsworth	18.3	Reasonable	Silwood	No
Blind_test_49	Harmondsworth	34.2	High	Harmondsworth	Yes
Blind_test_50	Rhosmaen	11.3	Reasonable	Rhosmaen	Yes
Blind_test_51	Harmondsworth	24.8	High	Lampeter	No
Blind_test_52	Rhosmaen	0.9	Low	Rhosmaen	Yes
Blind_test_54	Lampeter	10.9	Reasonable	Silwood	No
Blind_test_55	Lampeter	10.6	Reasonable	Lampeter	Yes

^a,b,c^Defined in “Methods” section

## Discussion

Invasive weeds are a significant economic problem throughout the world; a problem for which biological control (the use of co-evolved natural enemies isolated from the region of origin of the invasive weed) can be an effective and environmentally-sustainable solution. In the current study, we have focused on *I. glandulifera*; an invasive annual weed native to the foothills of the Himalayas for which two strains of a rust-based biological control agent are currently available (one originating from India and the other from Pakistan). As observed in previous weed biocontrol programmes, particularly when using plant pathogens, which are often intrinsically linked to their hosts, the susceptibility of some weed populations to the biocontrol agent can vary [19, 20]. A successful biocontrol programme is determined by a fully compatible and virulent host-plant interaction. Current practice for optimising such target weed-biocontrol agent pairings is empirical; target plants obtained from a particular site of interest are grown and tested for their susceptibility to the available rust strains. This can be a slow and relatively-expensive process for which a faster and cheaper alternative would clearly be economically advantageous. Given the exquisite resolving power of MALDI-TOF MS-based analysis and the proven track record of this technique in clinical microbiology [3–8], we sought to employ MALDI-TOF MS for the identification and/or characterisation of *I. glandulifera* and closely-related *Impatiens* spp. as well as regional biotypes of *I. glandulifera* with the ultimate aim of being able to match the optimal rust-based biological control agent with the observed regional biotype of *I. glandulifera* with a known susceptibility profile at a particular site of interest.

Whilst a number of excellent sample-preparation methods are currently available and detailed in the above references, as mentioned earlier, these methods are not particularly convenient for the analysis of plant material and so, in order to overcome this, we developed a highly-simplified and inexpensive method for MALDI-TOF MS sample preparation that is applicable to plant material in which cells are lysed by maceration in aqueous acetonitrile containing trifluoroacetic acid (TFA), which selectively extracts acid-soluble proteins. Lysis and extraction are carried out in the presence of near-saturated and inexpensive-grade MALDI matrix. The resulting matrix-saturated lysate containing acid-solubilised proteins is then dried down directly onto the MALDI-TOF MS sample plate, overlaid with additional matrix (if required), and analysed. Acidified acetonitrile was chosen as the solvent basis for our method, with the aim of aiding cell lysis by disruption of the membrane-stabilising hydrophobic effect using high concentrations of acetonitrile and selective ribosomal-protein solubilisation through low pH. TFA was chosen for acidification because it is a significantly stronger acid than formic acid, which is often used in MALDI-TOF MS sample-preparation methods [3]. This means that comparable proton concentrations can be obtained from much lower concentrations of acid, thereby dramatically reducing the amount of odorous material evaporating from the reagent during use. In many current methods (reviewed in [5]), after protein extraction, the acidic supernatant from formic acid treatment is dried down onto the sample plate prior to overlay with matrix solution. Once dried down onto solid surfaces many proteins often re-solubilise inefficiently. We therefore chose to premix the proteins and matrix and then dry these down *together*. In order to make this economically feasible, inexpensive matrix is necessary and so we decided to employ less-expensive grades of HCCA, with 98% pure C2020-10G supplied by Sigma being, for example, around 500 times cheaper per gram than many 99%-pure ‘MALDI-grade’ reagents. In direct-transfer protocols [5], highly-purified matrix is eventually mixed with a crude cell lysate so we reasoned that the importance of small differences in reagent purity is perhaps overstated given our intended usage. In addition, because it does not require centrifugation, our method is less constrained in terms of throughput and can more readily be employed in resource-poor settings. As we add plant material to a single optimised reagent, macerate, and then pipette one microlitre of the crude lysate onto the MALDI sample plate, our method is highly-simplified. Using 80 µl of a 500-fold cheaper matrix solution rather than one microlitre of conventional matrix still makes our method sixfold cheaper in terms of matrix costs. In absolute terms, our method takes around 2 min of labour time per sample (depending on how easy the plant material is to macerate) and has reagent costs of 1.2 UK pence per sample (0.8 UK pence for acetonitrile, 0.1 UK pence for TFA, and 0.3 UK pence for HCCA matrix).

Using the above method, we have been able to discriminate between the closely-related *Impatiens* spp., *I*. *noli*-*tangere*, *I. parviflora*, *I*. *scabrida*, *I*. *balsamina*, and *I*. *glandulifera* and between regional biotypes of *I*. *glandulifera*, and three iterations of experimental design and data analysis have been presented for sample discrimination.

In the first of these, we have used a combination of PCA and closest-relatedness diagrams. PCA allows for a simple two-dimensional visualisation of spectral relatedness. As the spectral similarity between samples increases however, we observe that PCA clustering converges to a point at which grouping of different regional biotypes within a species can be difficult to delineate (as observed for example in Fig. 2 above for the remaining samples not in PCA groups 1, 2, and 3). In order to circumvent this difficulty, we developed the method described above which is based upon closest-relatedness diagrams.

Once again, as the spectral similarity between samples increases, we observe that the closest-relatedness diagrams become more complex and difficult to resolve (as observed for example in strongest-match linkage group 5 in Table 2 above). The latter difficulty caused us to modify our approach to the method described above for discrimination by identification of test samples against reference-sample spectra. Whilst this method was initially shown to work with single leaves from single plants, we also employed identification of test samples against reference samples using multiple leaves from multiple plants in order to investigate the impact of leaf age on the discrimination between the regional biotypes of *I*. *glandulifera* described above.

By way of comparison, the methods using PCA and closest-relatedness diagrams have the advantage that they can be used for sample grouping without knowledge of or access to reference materials. The methods using discrimination by identification of test samples against reference-sample spectra are a little more constrained because of this but they are much more powerful at discrimination between samples that are very similar in terms of their MALDI-TOF MS spectra. They are also free of any subjective bias that is possible where groupings are made on the basis of closely-clustered PCA ordination plots.

Mehta and Silva [9] have previously discussed the potential problems for MALDI-TOF MS from very high abundance proteins such as ribulose bisphosphate carboxylase-oxidase (RUBISCO) that are associated with photosynthesis in plant tissues. For our sample-preparation method throughout these studies, we have been able to obtain mass spectra that contain numerous peaks and which are not overwhelmed by one or a small number of dominant protein peaks. We speculate that the reason for this is that our method selectively analyses only the acid-soluble fraction of proteins that are also soluble in 60–65% (v/v) acetonitrile and that RUBISCO and other such very high abundance proteins are not soluble under these conditions.

Having demonstrated identification of test samples against reference samples for the discrimination between the regional biotypes of *I*. *glandulifera* described above, this raises the possibility that further development of this technique might enable the identification of *I*. *glandulifera* regional biotypes to a level that could predict their susceptibility to available strains of Himalayan balsam rust; a capability that would significantly increase both the efficiency and efficacy of biological control of this invasive weed—and area of research that we intend to pursue in future studies. Preliminary molecular work based on DNA sequences of multiple chloroplast loci suggested that there are a number of different biotypes present in the UK but there is no clear link to rust susceptibility (D. Kurose, personal communication). In particular, it would be desirable to understand better the increase in spectral variance that is observed with younger leaves. As our method selectively extracts acid-soluble proteins for MALDI-TOF MS analysis, many of these will be high-abundance ribosomal proteins. Given that younger leaves are actively growing and undergoing significant development, it is possible that the observed MALDI-TOF MS proteome based upon acid-soluble proteins is sensitive to the developmental stage of the sampled tissue. Given the degree of closeness of the regional biotypes of *I*. *glandulifera* described above, it is possible that this adds sufficient variance compared to using older leaves for this to confound the differentiation between regional biotypes. Better understanding of this potential source of MALDI-TOF MS spectral variance will therefore be investigated following on from the work described in this paper.

## Conclusions

We have developed a highly-simplified and inexpensive method for MALDI-TOF MS sample preparation that is applicable to plant material in which cells are lysed by maceration in aqueous acetonitrile containing TFA, which selectively extracts acid-soluble proteins. Lysis and extraction are carried out in the presence of near-saturated and inexpensive-grade MALDI matrix. The resulting matrix-saturated lysate containing acid-solubilised proteins is then dried down directly onto the MALDI-TOF MS sample plate, overlaid with additional matrix (if required), and analysed. Mass spectra generated using this method do not appear to be overwhelmed by very high abundance proteins such as RUBISCO that are associated with photosynthesis in plant tissues. Using the above method along with a combination of principal-component analysis and closest-relatedness diagrams derived from MALDI-TOF MS spectral-comparison data, discrimination is possible between the closely-related *Impatiens* spp. *I. noli-tangere*, *I. parviflora*, *I. scabrida*, *I. balsamina*, and two regional biotypes of the invasive weed *I. glandulifera*. Using the above method in combination with sample discrimination based upon comparison between blind-test MALDI-TOF MS spectra and reference-sample spectra, discrimination is also possible, on the basis of the acid-soluble-protein mass spectra generated, between four regional biotypes of *I. glandulifera* that differ in their susceptibility to the biological control agent Himalayan balsam rust (*P. komarovii* var. *glanduliferae*) using mature leaf material. Using younger leaves, discrimination is not possible between these four regional biotypes. MALDI-TOF MS holds great promise for improving weed biological control through matching of biological control agents with susceptible target species/regional biotypes.

## Additional files

**Additional file 1: Figure S1.** Graphical representation of sampling for Experiment 1, in which one plant per species, one leaf per plant, and four replicate leaf fragments per leaf were employed.

**Additional file 2: Figure S2.** Graphical representation of sampling for Experiment 2, in which one plant per biotype, one leaf per plant, three replicate leaf fragments per leaf to make reference spectra, and four replicate leaf fragments per leaf to use for blind-testing against the reference spectra were employed.

**Additional file 3: Figure S3.** Graphical representation of sampling for Experiment 3, in which two plants per biotype, two leaves per plant (one old and one new), one leaf fragments per leaf to make reference spectra, and two replicate leaf fragments (A and B) per leaf each spotted twice (1 and 2) onto the MALDI plate for blind-testing against the reference spectra were employed.

## References

[1] Karas M, Bachmann D, Hillenkamp F (1985). Influence of the wavelength in high-irradiance ultraviolet laser desorption mass spectrometry of organic molecules. Anal Chem.

[2] Knochenmuss R (2006). Ion formation mechanisms in UV-MALDI. Analyst.

[3] Cassagne C, Ranque S, Normand AC, Fourquet P, Thiebault S, Planard C, Hendrickx M, Piarroux R (2011). Mould routine identification in the clinical laboratory by matrix-assisted laser desorption ionization time-of-flight mass spectrometry. PLoS ONE.

[4] Bader O (2013). MALDI-TOF-MS-based species identification and typing approaches in medical mycology. Proteomics.

[5] Clark AE, Kaleta EJ, Arora A, Wolk DM (2013). Matrix-assisted laser desorption ionization–time of flight mass spectrometry: a fundamental shift in the routine practice of clinical microbiology. Clin Microbiol Rev.

[6] Adams LL, Salee P, Dionne K, Carroll K, Parrish N (2015). A novel protein extraction method for identification of mycobacteria using MALDI-ToF MS. J Microbiol Methods.

[7] Fraser M, Brown Z, Houldsworth M, Borman AM, Johnson EM (2016). Rapid identification of 6328 isolates of pathogenic yeasts using MALDI-ToF MS and a simplified rapid extraction procedure that is compatible with the Bruker Biotyper platform and database. Med Mycol.

[8] Singhal N, Kumar M, Kanaujia PK, Virdi JS (2015). MALDI-TOF mass spectrometry: an emerging technology for microbial identification and diagnosis. Front Microbiol.

[9] Mehta A, Silva LP (2015). MALDI-TOF MS profiling approach: how much can we get from it?. Front Plant Sci.

[10] Coombe DE (1956). Notes on some British plants seen in Austria. Veröffentlichungen des Geobotanisches Institut.

[11] Tanner R, Ellison C, Shaw R, Evans H, Gange A (2008). Losing patience with *Impatiens*: are natural enemies the solution?. Outlook Pest Manag.

[12] Tanner RA, Ellison CA, Seier MK, Kovács GM, Kassai-Jáger E, Berecky Z, Varia S, Djeddour D, Singh MC, Csiszár Á, Csontos P, Kiss L, Evans HC (2014). *Puccinia komarovii* var. *glanduliferae* var. nov.: a fungal agent for the biological control of Himalayan balsam (*Impatiens glandulifera*). Eur J Plant Pathol.

[13] Tanner RA, Pollard KM, Varia S, Evans HC, Ellison CA (2015). First release of a fungal classical biocontrol agent against an invasive alien weed in Europe: biology of the rust *Puccinia komarovii* var. *glanduliferae*. Plant Pathol.

[14] Hatcher PE (2003). Biological flora of the British Isles: *Impatiens noli*-*tangere* L. J Ecol.

[15] Shaw RH, Ellison CA, Marchante H, Pratt CF, Schaffner U, Sforza RFH, Deltoro V (2015). Weed biological control in the European Union: from serendipity to strategy. BioControl.

[16] Varia S, Pollard K, Ellison C (2016). Implementing a novel weed management approach for Himalayan balsam: progress on biological control in the UK. Outlook Pest Manag.

[17] Nagy A-M, Korpelainen H (2015). Population genetics of Himalayan balsam (*Impatiens glandulifera*): comparison of native and introduced populations. Plant Ecol Div.

[18] Janssens S, Geuten K, Yuan Y-M, Song Y, Kupfer P, Smets E (2006). Phylogenetics of *Impatiens* and *Hydrocera* (Balsaminaceae) using chloroplast *atpB*-*rbcL* spacer sequences. Syst Botany.

[19] Hasan S, Ayres PG (1990). The control of weeds through fungi: principles and prospects. New Phytol.

[20] Ellison CA, Evans HC, Ineson J. The significance of intraspecies pathogenicity in the selection of a rust pathotype for the classical biological control of *Mikania micrantha* (mile-a-minute weed) in Southeast Asia. 2004: Proceedings of the XI International Symposium on Biological Control of Weeds, Canberra, Australia, 27 April–2 May, 2003 2004 p. 102–107 ref.16.

