# Supplementary material for: Differentiation between closely-related Impatiens spp. and regional biotypes of Impatiens glandulifera using a highly-simplified and inexpensive method for MALDI-TOF MS
Source: Plant Methods. 2018 Jul 16;14:60. doi: 10.1186/s13007-018-0323-6 (PMC6047133; doi:10.1186/s13007-018-0323-6)
Supplement: Supplementary file 1 — Additional file 1: Figure S1. Graphical representation of sampling for Experiment 1, in which one plant per species, one leaf per plant, and four replicate leaf fragments per leaf were employed. [file 13007_2018_323_MOESM1_ESM.docx]

**Supplementary Figure S1** Graphical representation of sampling for Experiment 1, in which one plant per species, one leaf per plant, and four replicate leaf fragments per leaf were employed.
