# Supplementary material for: Differentiation between closely-related Impatiens spp. and regional biotypes of Impatiens glandulifera using a highly-simplified and inexpensive method for MALDI-TOF MS
Source: Plant Methods. 2018 Jul 16;14:60. doi: 10.1186/s13007-018-0323-6 (PMC6047133; doi:10.1186/s13007-018-0323-6)
Supplement: Supplementary file 2 — Additional file 2: Figure S2. Graphical representation of sampling for Experiment 2, in which one plant per biotype, one leaf per plant, three replicate leaf fragments per leaf to make reference spectra, and four replicate leaf fragments per leaf to use for blind-testing against the reference spectra were employed. [file 13007_2018_323_MOESM2_ESM.docx]

**Supplementary Figure S2** Graphical representation of sampling for Experiment 2, in which one plant per biotype, one leaf per plant, three replicate leaf fragments per leaf to make reference spectra, and four replicate leaf fragments per leaf to use for blind-testing against the reference spectra were employed.
