# Supplementary material for: Differentiation between closely-related Impatiens spp. and regional biotypes of Impatiens glandulifera using a highly-simplified and inexpensive method for MALDI-TOF MS
Source: Plant Methods. 2018 Jul 16;14:60. doi: 10.1186/s13007-018-0323-6 (PMC6047133; doi:10.1186/s13007-018-0323-6)
Supplement: Supplementary file 3 — Additional file 3: Figure S3. Graphical representation of sampling for Experiment 3, in which two plants per biotype, two leaves per plant (one old and one new), one leaf fragments per leaf to make reference spectra, and two replicate leaf fragments (A and B) per leaf each spotted twice (1 and 2) onto the MALDI plate for blind-testing against the reference spectra were employed. [file 13007_2018_323_MOESM3_ESM.docx]

**Supplementary Figure S3** Graphical representation of sampling for Experiment 3, in which two plants per biotype, two leaves per plant (one old and one new), one leaf fragments per leaf to make reference spectra, and two replicate leaf fragments (A and B) per leaf each spotted twice (1 and 2) onto the MALDI plate for blind-testing against the reference spectra were employed.
